# Built and Social Environmental Correlates of Brain and Behavioral Health Among Youth

**DOI:** 10.3390/healthcare14213737

**Published:** 2026-11-03

**Authors:** Xiangli Gu, Caifang Wu, Zhibin Lin, Hong Pan, Mira Ghraizi, Tao Zhang

**Affiliations:** 1 Department of Kinesiology, The University of Texas at Arlington, Arlington, TX 76019, USA; caifang.wu@uta.edu (C.W.); mira.ghraizi@uta.edu (M.G.); 2 Department of Civil Engineering, The University of Texas at Arlington, Arlington, TX 76019, USA; zhibin.lin@uta.edu (Z.L.); hong.pan@uta.edu (H.P.); 3 Department of Kinesiology, Health Promotion and Recreation, University of North Texas, Denton, TX 76203, USA; tao.zhang@unt.edu

**Keywords:** built environment, cognitive function, behavioral health

## Abstract

**Highlights:**

**What are the main findings?**

- Presents an ecological framework linking place-based environmental factors with physical activity and cognitive health in youth.
- Demonstrates built and social environmental pathways for promoting youth health.

**What are the implications of the main findings?**

- Highlights the importance of neighborhood safety and the home media environment in shaping healthy behaviors.
- Provides a replicable framework for examining place-based environmental influences on health outcomes.

**Abstract:**

Background/Objectives: Guided by the Social Ecological Model, this study examined the relationships between ecological environmental factors, executive function, physical activity, and sedentary behavior among youth. Methods: A cross-sectional study was conducted among 283 youth (M_age_ = 15 years; Male = 33.6%) in the North Texas region. Parent-report surveys were used to assess ecological environmental factors, executive function, physical activity, and sedentary behavior. Sociodemographic characteristics, including household income, ethnicity, age, and sex were also collected. Results: Neighborhood safety/crime was significantly associated with all components of executive function (*p* < 0.01). Community walkability was significantly associated with overall executive function and planning (β = 0.16 and 0.18, *p* < 0.05) and with screen-based sedentary behavior (r = −0.23, *p* < 0.01). Neighborhood safety/crime and the home media environment significantly predicted physical activity (β = −0.34; β = −0.29, *p* < 0.01; R^2^ = 20.4%), whereas the socioeconomic environment emerged as a significant predictor of daily sedentary behavior (β = 0.16; *p* < 0.05; R^2^ = 18.1%). The mediation model showed acceptable fit, chi-square (df = 25) = 420.32, *p* = 0.017, CFI = 0.956, and RMSEA = 0.050, 90% CI [0.021, 0.075]. The direct path from environmental factors to executive function was statistically significant (*p* < 0.01); however, the indirect effect of environmental factors on executive function through physical activity was not supported. Conclusions: These findings suggest that neighborhood safety and walkability are associated with executive function in school-aged youth, independent of physical activity levels. Because the data are cross-sectional, these implications should be tested in longitudinal and intervention studies before informing clinical guidelines.

## 1. Introduction

The World Health Organization (WHO) defines brain health as the state of brain functioning across cognitive, sensory, social–emotional, behavioral, and motor domains that allows a person to realize their full potential over the life course, irrespective of the presence or absence of disorders [1]. This functional definition extends beyond the absence of neurological disease, and conceptual analyses similarly describe brain health as a multidimensional construct [2]. Cognitive functioning is one domain within this broader construct and refers to mental processes, such as attention, memory, and reasoning, through which the brain supports learning and behavior. Executive function (EF) comprising working memory, planning, inhibitory control, and cognitive flexibility, represents one of the most consequential domains of cognitive development in school-aged youth [3]. These top-down regulatory processes govern goal-directed behavior, resistance to impulse, and the capacity to adapt flexibly to complex, rapidly changing environments [3,4]. Because the prefrontal cortex as the primary neural substrate of EF undergoes protracted maturation through late adolescence and into early adulthood [5], this developmental period is uniquely sensitive to both supportive and adverse environmental conditions. Deficits in EF during adolescence are strongly associated with attention-deficit/hyperactivity disorder (ADHD), poor academic achievement, elevated health-risk behavior, and compromised occupational outcomes [6], establishing EF as a priority target for public health research. It is important to understand how the environments in which adolescents live including the built environment, community infrastructure, residential location, and socioeconomic circumstances shape EF development, which also emerges urgent implications for community planning, public health policy, and educational practice.

A compelling and growing body of evidence notes physical activity (PA) as one of the most robust modifiable behavioral mechanisms linking environmental conditions to brain health in youth. Research documented that moderate-to-vigorous physical activity (MVPA) promotes EF through well-characterized neurobiological pathways, including increased cerebral blood flow, hippocampal neurogenesis, and upregulation of brain-derived neurotrophic factor (BDNF) in the prefrontal cortex [7]. Systematic reviews and meta-analyses confirm that both acute and chronic exercise yield significant improvements in working memory, inhibitory control, and cognitive flexibility in children and adolescents [8,9]. Conversely, sedentary behavior (SB), particularly recreational screen-based SB, is negatively associated with EF, most consistently for inhibitory control and cognitive flexibility [10]. Despite this well-established behavioral link between PA, SB, and EF, the literature remains unclear on what features of the ecological and built environment enable or constrain these associations.

The Social Ecological Model (SEM) [11,12] provides theoretical architecture for this investigation. The SEM posits that health behavior is shaped by multilevel influences including individual, interpersonal, organizational, community, and policy, that operate simultaneously and interactively. The SEM literature has demonstrated that community-level factors, including neighborhood safety, built environment design, recreational infrastructure, and socioeconomic conditions, are as consequential as individual-level determinants in predicting youth PA participation [13,14]. A multilevel meta-analysis by Zhao and colleagues [15] confirmed that community and environmental factors exerted significant independent effects on PA participation in children and adolescents across multiple nations. Smith and colleagues [16], in a systematic review linking built environment to PA and active transport, found consistent positive effects of walkability, park quality, and pedestrian infrastructure on PA, with effects moderated by socioeconomic status, underscoring the equity implications of built environment design for physical health outcomes. However, no study to date has integrated multiple ecological-level factors within a single model to simultaneously examine their effects on both PA behavior and EF in adolescents.

### 1.1. Built Environment and Pedestrian Infrastructure

The built environment encompassing neighborhood design, street connectivity, land use mix, residential density, and access to recreational facilities, constitutes the physical substrate within which adolescents’ daily movement behavior is embedded. Community walkability, derived from geospatial assessments of street network connectivity, destination density, and sidewalk continuity, is a structural property of urban and suburban infrastructure with well-documented effects on youth PA. Sallis et al. [17] found that adolescents in high-walkability neighborhoods accumulated significantly more transport walking and total PA, and spent less time in SB, relative to peers in low-walkability areas. Caetano et al. [18], using kernel density estimation and GIS-derived walkability indices in a community sample of 292 adolescents, demonstrated that proximity to PA-enabling destinations and higher walkability scores were associated with significantly greater odds of meeting PA guidelines. Loh et al. [19], using GIS-based street network buffers in a multilevel study, confirmed that walkability components, particularly land use mix and residential density, were positively associated with accelerometer-measured MVPA outside school hours, with safety perceptions moderating these built environment effects. The pedestrian infrastructure of a community directly structures the opportunity and safety conditions for active transport, outdoor play, and unstructured physical activity, the very behaviors that sustain prefrontal cortical health during development [7,20]. Integrating geospatial walkability measures alongside other ecological indicators is therefore essential for a comprehensive model of environmental influences on adolescent PA and EF.

### 1.2. Neighborhood Safety and Crime Exposure

Perceived and objective neighborhood safety represent critical community-level factors that moderate the built environment’s influence on PA. When adolescents or their caregivers perceive neighborhoods as unsafe due to crime, traffic, or community violence, opportunities for outdoor PA are constrained by parental restrictions on independent mobility and by the adolescents’ own fear-based avoidance of outdoor spaces [21]. A nationally representative cohort study of 11,726 UK adolescents found that both perceived neighborhood safety and objective crime index scores were significantly associated with PA, with fear of crime functioning as a barrier particularly for girls [21]. Beyond its suppression of PA, neighborhood safety also exerts direct neurobiological effects on EF. According to the data from the Adolescent Brain Cognitive Development (ABCD) Study, Lindsley and colleagues [22] found that perceived neighborhood safety was associated with adolescent cognitive performance and executive control. Chronic threat perception activates the hypothalamic–pituitary–adrenal (HPA) axis, elevating cortisol and constraining the neuroplasticity of the prefrontal cortex and hippocampus, regions most central to working memory, planning, and inhibitory control [22,23]. These converging behavioral and neurobiological pathways make neighborhood safety a foundational variable in any ecologically comprehensive model of adolescent EF.

### 1.3. Socioeconomic Environment and Household Income

Socioeconomic environment indexed by household income, area deprivation, and access to economic resources shape both PA behavior and EF through multiple, interacting pathways. Lower household income constrains access to safe recreational spaces, organized sports programs, and activity-enabling built environments, producing systematic disparities in MVPA and SB across socioeconomic strata [24]. Mesquita et al. [25] demonstrated that parent–child physical activity associations were strongest in low-income households, where modeling of sedentary behavior was also most pronounced. Beyond its behavioral effects, socioeconomic deprivation exerts a direct neurological burden on EF development, that is, poverty-associated chronic stress reduces gray matter volume in the dorsolateral prefrontal cortex and anterior cingulate cortex, disrupts prefrontal synaptic architecture, and alters amygdala-prefrontal functional connectivity in ways that impair cognitive regulation [26]. Using data from the ABCD Study, Dennis and colleagues [27] confirmed that household income was independently associated with EF performance and prefrontal cortical volume, with effects partially mediated by BMI, underscoring the co-occurring physical and cognitive health disparities that track socioeconomic disadvantages. These findings establish socioeconomic environment as both behavioral and neurobiological determinant of adolescent EF, operating through which mechanism remains unknown.

### 1.4. Geographic Environment/Residential Context

Geographic environment operationalized through residential ZIP code, urban–suburban–rural classification, and area-level indicators of resource concentration and environmental quality represent the broadest structural stratum of ecological influence on adolescent health behavior. ZIP code has been described as one of the strongest predictors of health outcomes in the United States, capturing the accumulated burden of socioeconomic disadvantage, environmental hazard exposure, and built environment quality that cluster geographically [20]. GIS-derived analyses have consistently demonstrated that residential location predicts access to walkable streets, parks, recreational infrastructure, and safe outdoor spaces that enable PA [18,19]. At the neurodevelopmental level, neighborhood disadvantage measured by area deprivation indices has been associated with reduced cortical surface area in the prefrontal cortex, altered amygdala-prefrontal connectivity, and lower performance on EF assessments in children and adolescents from the ABCD Study cohort [22,27]. These geospatial effects on brain development underscore the importance of including residential location, not simply as a covariate, but as an analytically meaningful ecological variable.

This study tested interdisciplinary framing to provide preliminary evidence on which ecological conditions most powerfully constrain adolescent behaviors and brain health and identifying the community infrastructure targets most amenable to intervention by public health practitioners, civil engineers, and urban planners alike. Neighborhood environments are not distributed at random. Residential segregation and structural racism concentrate crime, limited recreational resources, and environmental stressors in low-income communities and communities of color, making place of residence a fundamental cause of health disparities [28,29]. From an environmental justice perspective, unsafe or under-resourced neighborhoods are upstream determinants that can shape children’s development through several routes simultaneously, rather than through a single behavioral pathway. How the environment is measured also shapes what it can explain. Perceived and objective environmental measures capture related but distinct constructs [30,31]. For children, parents’ perceptions may be especially consequential, because parents act as gatekeepers who permit or restrict outdoor play according to how safe they judge the neighborhood to be. PA is one plausible pathway linking the environment to executive function, but other pathways are also likely, including the socioeconomic disadvantage and exposure to neighborhood violence [32,33]. The existing literature supports a robust evidence base for individual links among ecological environment; however, no published study has simultaneously examined the effects of multiple ecological factors on PA behaviors and EF components within a single integrated model. We therefore conceptualized ecological environmental factors as operating through multiple parallel pathways, as well as through direct associations with executive function.

Guided by the Social Ecological Model [12], the purpose of this study was to examine the relationships of ecological environmental factors, including neighborhood safety/crime, community walkability, home media environment, socioeconomic environment (i.e., household income), and geographic environment (i.e., ZIP code or residential location), with EF (i.e., working memory [WM], planning [PL], regulation [RE] and inhibition [IN]), and physical activity (PA; light PA [LPA], moderate-to-vigorous PA [MVPA]) and sedentary behavior (SB; screen-based SB and non-screen-based SB) among school-aged youth; and to test the potential mediating role of physical activity in the relationship between ecological environmental factors and executive function among youth.

## 2. Materials and Methods

### 2.1. Participants and Setting

A cross-sectional research design was implemented and a total of 283 parents/guardians of school-aged youth were recruited with most participants coming from the metropolitan areas of the United States. Each parent/guardian reported on one target child. The youth in this study had a mean age of 15 years (SD = 3.2 years), 33.6% were male, and 35.1% were identified as Hispanic. Age data were available for 244 participants; 39 had missing age data. Most participants were adolescents: 225 (92.2%) were aged 13–18 years, 17 (7.0%) were aged 11–12 years, and 2 (0.8%) were younger than 11 years.

Participants were recruited from various community events in the North Texas region, including school sport events and afterschool programs in local schools. Eligible participants were parents or legal guardians of a child aged 7–18 years who were able to complete the survey in English or Spanish. Surveys without informed consent were excluded. Data was collected through parent-reported surveys. Body mass index (BMI) was calculated using parent-reported height and weight and was used as a continuous covariate in the analyses. The full school-aged range was retained because the Social Ecological Model proposes that home, neighborhood, and community environments shape movement behaviors and EF across childhood and adolescence, and because the sample was predominantly adolescent. Child age was included as a covariate in all analyses.

The study was approved by the university institutional review board. After IRB approval, digital flyers with an embedded QuestionPro survey link were distributed as the primary recruitment method. Through the survey link, parents/guardians were able to review and sign informed consent before beginning the online survey. Participants who did not provide consent were automatically withdrawn from the study. All participants were fully informed about the purpose, procedures, risks, and benefits of the study before agreeing to participate. Participants were also informed that participation was voluntary and that they could refuse or discontinue participation at any time without penalty or loss of benefits.

### 2.2. Ecological Environmental Factors

Socioeconomic environment: The socioeconomic environment was assessed using parent-reported household income from the parent survey. Household income was used as an indicator of the family’s socioeconomic context, reflecting available economic resources that may influence children’s access to PA opportunities and resources.

Geographic environment: The geographic environment was assessed using parent-reported residential ZIP code from the parent survey. ZIP code was used to characterize participants’ residential location and broader geographic context, which may be linked to neighborhood-level environmental conditions and community resources, and was not analyzed as a numeric value [34]. Each ZIP code was matched to its primary 2020 Rural–Urban Commuting Area (RUCA) code from the U.S. Department of Agriculture Economic Research Service, which classifies areas by population density, urbanization, and daily commuting patterns [35]. RUCA codes were recorded into four ordered categories: 1 = metropolitan core (RUCA 1), 2 = metropolitan high commuting (RUCA 2), 3 = micropolitan core (RUCA 4), and 4 = rural (RUCA 10), with higher values indicating more rural residence. Most participants (98.2%) resided in metropolitan-core areas (Table 1).

Home media environment: The home media environment was assessed using items adapted from the Neighborhood Impact on Kids (NIK) study parent survey [36]. Parents reported whether each of seven types of electronic devices was available in their child’s bedroom, including televisions, computers, game consoles, and cell phones. Each item was coded 1 (yes) or 2 (no), and items were summed (range = 7–14), with higher scores indicating fewer media devices in the child’s bedroom. Reliability of the home media environment scale was moderate in the present sample, with an average-measures intraclass correlation coefficient (two-way mixed, consistency) of 0.57 (95% CI [0.48, 0.65]) [37,38]. This domain captures the availability of bedroom-based media devices, which may contribute to increased screen time, SB, and shorter sleep duration.

Community walkability environment: Community walkability was assessed using the parent version of the Neighborhood Environment Walkability Survey for Youth (NEWS-Y) [39]. Parents completed 5 items assessing perceptions of neighborhood design and safety features that may support active travel and outdoor activity, including street connectivity, availability of alternative routes, adequacy of street lighting, and perceived safety from crime or stranger danger. Items were rated on a 4-point scale (1 = strongly disagree to 4 = strongly agree) and summed (range = 5–20), with higher scores indicating more supportive walking environments. The NEWS-Y parent and youth versions have shown test–retest reliability and associations with youth PA [39]; the parent version was evaluated among parents of children aged 5–11 years and of adolescents aged 12–18 years, which covers the age range of the present sample. Parent-reported perceptions of neighborhood walkability and safety have also shown acceptable construct validity (confirmatory factor analysis) and associations with PA in a large sample of U.S. youth aged 3–17 years [40].

Neighborhood safety/crime environment: Neighborhood safety and crime were assessed using items adapted from the Neighborhood Impact on Kids (NIK) questionnaire. Parents completed 9 items regarding perceived barriers to their child’s outdoor play and PA in nearby parks, playgrounds, or neighborhood streets, including lack of equipment, supervision, or peers, as well as concerns about crime, traffic, and poor lighting. Parents also reported whether they or someone they knew had been a victim of crime in the neighborhood. This domain reflects parents’ perceptions of neighborhood safety and social conditions that may limit children’s opportunities for outdoor play and PA within the community.

### 2.3. Physical Activity and Sedentary Behavior

Physical activity: The Godin–Shephard leisure-time physical activity questionnaire [41] was used to assess individuals’ participation in PA in their leisure time. Parents reported their child’s frequency of participating in vigorous–moderate and mild/light physical activities, respectively for more than 15 min in free time in the past 7 days. Based on the scoring guideline [42], LPA was calculated as LPA = mild frequency × three metabolic equivalents of task (MET); MVPA was calculated as MVPA = moderate frequency × five MET + strenuous frequency × nine MET, respectively. The calculated index for LPA and MVPA were used in the data analysis. The Godin–Shephard questionnaire has shown acceptable test–retest reliability among children and adolescents (r = 0.81) [42].

Sedentary behavior: Screen-based SB and non-screen-based SB were measured according to the amount of time engaged in eight sitting behaviors by the Sedentary Behavior Questionnaire (SBQ) [43]. The SBQ includes screen-based SB (i.e., sitting while watching television, using the computer for study, playing on computer/video games, and talking/texting on the phone) and non-screen-based SB (i.e., sitting while listening to music, doing paperwork or office work, reading and doing arts or crafts, and driving/taking cars or bus). Each sitting behavior was scaled in nine-time frames per day (i.e., 0, ¼, ½, 1, 2, 3, 4, 5, 6 and above 6 h). The sum of items from the screen-based and non-screen-based behaviors was used in the final data analysis.

### 2.4. Executive Functions

The Childhood Executive Function Inventory (CHEXI) for Parents & Teachers was used to assess EF [44]. The CHEXI includes 24 items across four domains: working memory (9 items), planning (4 items), regulation (5 items), and inhibition (6 items). Example items include “My child has difficulty remembering lengthy instructions” for working memory, “My child has difficulty with tasks or activities that involve several steps” for planning, “My child has clear difficulties doing things he/she finds boring” for regulation, and “My child has a tendency to do things without first thinking about what could happen” for inhibition. Parents rated each item on a 5-point scale ranging from 1 = *Not true* to 5 = *Definitely true*. Higher scores indicate greater EF difficulties, reflecting lower EF. The internal consistency of the CHEXI in the present study was excellent, with a Cronbach’s alpha of 0.90. The CHEXI used in the present study was developed specifically to assess EFs in everyday contexts and has demonstrated adequate reliability and validity in parent and teacher reports [44]. Parent-reported EF therefore provides a valid and practical behavioral indicator of the cognitive domain of brain health in population-based research [45,46].

### 2.5. Statistical Analysis

The data analyses were processed in the SPSS Version 30.1.1 (Armonk, NY, USA: IBM Corp.). The descriptive analyses including mean and standard deviation were calculated. Pearson product-moment correlation was used to examine the bivariate relationships among study variables. Then three hierarchical linear regression models were processed on PA, SB, and EF; another four models were further processed on working memory, planning, inhibition and regulation. Sex, age, and ethnicity were controlled in the regression models as confounding variables. Regression assumptions were examined for each model. Multicollinearity was evaluated with variance inflation factors (VIF) and tolerance, independence of residuals with the Durbin–Watson statistic, normality with residual skewness and kurtosis, and homoscedasticity with the Breusch-Pagan test. Influential observations were identified with Cook’s distance and leverage.

Two multivariate analyses of covariance (MANCOVA) were conducted to examine the effects of sex (boys vs. girls) and race (Hispanic vs. Non-Hispanic) on PA, SB, and EF by controlling age and BMI. For the MANCOVA, partial eta squared (*ηp*^2^) with 90% confidence intervals was computed for each univariate effect [47], and values of 0.01, 0.06, and 0.14 were interpreted as small, medium, and large effects, respectively [48,49]. Because eight outcomes were tested for each grouping factor, the Holm–Bonferroni procedure was applied within each factor to control the familywise error rate [50]. Sex and ethnic comparisons were treated as exploratory analyses [51]. For the hierarchical regression models, 95% confidence intervals are reported for all unstandardized coefficients. SPSS AMOS 31 were performed to test the combined effects of the multiple ecological-level factors on both PA behavior and EF in adolescents. Specifically, a structural equation model was further estimated to examine the mediation pathway from the ecological environmental factors to EF through PA. Model fit was evaluated with χ^2^, χ^2^/df, CFI, TLI, and RMSEA with its 90% confidence interval. Conventional cutoffs (CFI and TLI ≥ 0.90; RMSEA ≤ 0.08) were treated as guidelines rather than strict criteria [52,53].

### 2.6. Sample Size and Missing Data

Participants were recruited through community events, school sport events, and afterschool programs; and no a priori power analysis was conducted. A sensitivity power analysis in G*Power 3.1 [54] indicated that the smallest regression sample provided 80% power at α = 0.05 to detect an increment of f2 ≥ 0.08 for the five environmental predictors with three covariates. The SEM estimated 29 free parameters with N = 283, approximately 10 cases per parameter [55,56]; power for the RMSEA test of close fit was 0.69 [57]. The mediation analysis was therefore treated as exploratory. The SEM was estimated with full-information maximum likelihood, which uses all available data under the missing-at-random assumption [58]. Bivariate correlations used pairwise deletion, and the regression and MANCOVA models used listwise deletion. Little’s MCAR test was significant, χ^2^(477) = 559.66, *p* = 0.005 [59]; however, participants included in the analytic samples did not differ from excluded participants in age, sex, ethnicity, or BMI (all *p* ≥ 0.32). Finally, a sensitivity analysis was conducted in which the SEM was re-estimated with the five environmental domains entered as separate, correlated observed predictors of PA and EF, with PA retained as a latent factor (full-information maximum likelihood; Supplementary Table S1). This specification does not assume that the environmental domains share a common underlying construct.

## 3. Results

Descriptive statistics for eco-environmental factors, PA, and EF outcomes are summarized in Table 1. Most participants (98.2%) lived in metropolitan core areas based on the RUCA classification of their residential ZIP code.

On average, participants spent around 2.28 h (SD = 1.77) on screen-based SB and 5.54 h (SD = 4.14) on non-screen-based SB per day, respectively. The descriptive results indicated that 26.9% of adolescents were classified as insufficiently active (MET-weighted MVPA score < 14) [40] in this study. Participants’ average CHEXI scores were 14.28 (SD = 6.22), 6.13 (SD = 3.11), 8.82 (SD = 3.59), 10.03 (SD = 3.79), and 39.30 (SD = 15.16) on working memory, planning, regulation, inhibition, and overall executive function, respectively, with higher scores indicating greater difficulties. The large standard deviations of executive function outcomes indicated wide variation across participants. However, CHEXI has no normative data, so no cut-off values are available to classify executive function deficits.

The results of bivariate correlation analysis are presented in Table 2. MVPA but not LPA was significantly associated with all four EF subscales including working memory (*r* = −0.16, *p* < 0.05), planning (*r* = −0.17, *p* < 0.05), regulation (*r* = −0.28, *p* < 0.01), and inhibition (*r* = −0.14, *p* < 0.05). Both MVPA and LPA were significantly associated with daily screen-based SB (*r* = −0.15 and *r* = −0.14, *p* < 0.05, respectively). Screen-based SB was significantly related to neighborhood safety/crime, and community walkability (*r*s ranging from −0.16 to −0.23, *p* < 0.05), whereas non-screen-based SB were not significantly associated with any geographic environment factors. The ecological environment factors were significantly related to different EF subscales in this study. Specifically, both neighborhood safety/crime and community walkability were significantly associated with all components of EF (*rs* range from 0.16 to 0.42, *p* < 0.05). Socioeconomic environment was associated with working memory, planning, and inhibition (*rs* = −0.13 to −0.21, *p* < 0.05), geographic environment was associated with planning (*r* = 0.18, *p* < 0.01) and regulation (*r* = 0.15, *p* < 0.05), and home media environment was associated with planning (*r* = −0.14, *p* < 0.05). The correlation analyses also showed that home media environment and neighborhood safety/crime were significantly associated with MVPA (*r_home_* = −0.24; r_neighborhood_ = −0.23; *p* < 0.01), but not with LPA.

Diagnostics indicated no multicollinearity (VIF ≤ 1.33; tolerance ≥ 0.75) and no observation with Cook’s distance greater than 1. The results of hierarchical regression models of ecological environment in predicting overall PA, SB and EF are presented in Table 3 and Table 4a,b. The results of the regression analysis showed that neighborhood safety/crime and home media environmental were significant predictors of daily PA (β = −0.34; β = −0.29; R^2^ = 20.4%, *p* < 0.01; Table 3). After control for the demographic variables (i.e., sex, age, ethnicity), socioeconomic environment emerged as a significant predictor of daily overall SB (β = 0.16, R^2^ = 18.1%, *p* < 0.05; Table 3).

Furthermore, the EF was significantly predicted by the geographic environment, neighborhood safety/crime, and community walkability (β = 15, β = 0.16, β = 0.27, *p* < 0.05; Table 3), the model explained 27.4% of the variance. To further explore the predictive utility of the ecological environment factors in EF, additional regression analyses were further conducted to examine its associations with specific EF subscales, including working memory, planning, regulation, and inhibition.

The regression models (Table 4a,b) show that neighborhood safety/crime environment consistently predicted all subscales of EF including working memory (β = 0.26, R^2^ = 28.9%; *p* < 0.01), planning (β = 0.24, R^2^ = 30.5%; *p* < 0.01), regulation (β = 0.31, R^2^ = 20.4%; *p* < 0.01), and inhibition (β = 0.23, R^2^ = 22.6%; *p* < 0.01), after controlling for all covariates (i.e., age, sex, and ethnicity).

**Figure 1:**
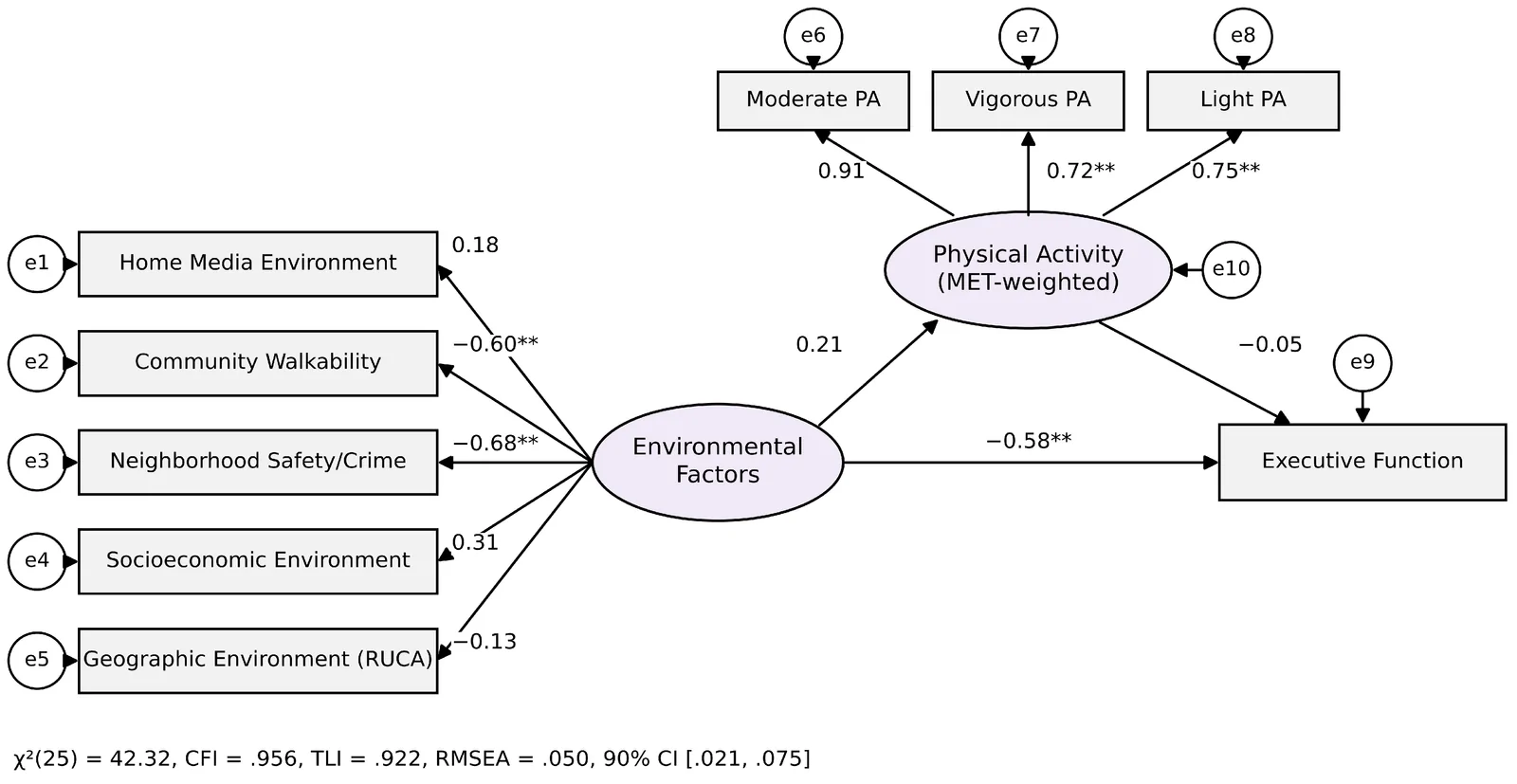
The Structural Equation Model for Social-Ecological Pathway; PA = physical activity. ** *p* < 0.01.

A structural equation model was further structured to examine the social-ecological pathway from the ecological environmental factors to EF by testing the mediation role of PA in this relationship (Figure 1). The ecological environmental factor was indicated by socioeconomic environment, geographic environment, home media environment, neighborhood safety/crime, and community walkability. PA was modeled as a latent factor indicated by LPA, MPA, and VPA. EF was included as an observed variable in the model. The model was estimated with full-information maximum likelihood and demonstrated acceptable fit, chi-square (df = 25) = 42.32, *p* = 0.017, Chi-square/df = 1.69, CFI = 0.956, IFI = 0.959, TLI = 0.922, NFI = 0.904, and RMSEA = 0.050, 90% CI [0.021, 0.075]. The structural path from the environmental factor to PA was positive but not significant (B = 5.377, SE = 2.971, β = 0.207, *p* = 0.07). The direct path from the environmental factor to EF was statistically significant (B = −27.96, SE = 8.732, β = −0.575, *p* < 0.001), whereas the direct path from PA to EF was not significant (β = −0.011, Monte Carlo 95% CI for the unstandardized effect [−2.60, 1.03]), and the standardized total effect was β = −0.586. The unstandardized and standardized SEM path coefficients are presented in Table 5.

In the sensitivity analysis with the environmental domains entered as separate predictors (Supplementary Table S1), the model fit the data well, χ^2^(12) = 10.61, *p* = 0.563, CFI = 1.00, RMSEA = 0.00. Neighborhood safety/crime was associated with PA (β = −0.23, *p* = 0.002) and EF (β = 0.30, *p* < 0.001), community walkability with EF (β = 0.20, *p* = 0.005), and the home media environment with PA (β = −0.29, *p* < 0.001). PA was not associated with EF (β = −0.09, *p* = 0.184), and no indirect effect through PA was significant.

Two 2 (sex) × 2 (ethnicity) MANCOVAs were further conducted to examine the effects of sex and ethnicity on PA and SB (Model 1) and on EF (Model 2), controlling for age and BMI (Table 6). The first MANCOVA model indicated a significant multivariate effect of sex [Wilks’ Λ = 0.949, *F* (4, 197) = 2.66, *p* = 0.034, *ηp*^2^ = 0.051]. However, univariate tests showed no significant sex differences in LPA *F* (1, 200) = 1.11, *p* = 0.293, *ηp*^2^ = 0.006], MVPA *F* (1, 200) = 0.25, *p* = 0.618, *ηp*^2^ = 0.001], screen-based SB *F* (1, 200) = 1.67, *p* = 0.198, *ηp*^2^ = 0.008], or non-screen-based SB [*F* (1, 200) = 1.39, *p* = 0.240, *ηp*^2^ = 0.007], indicating that the multivariate effect reflected a combination of small differences rather than a difference in any single outcome. The second MANCOVA model revealed no significant multivariate effect of sex on EF [Wilks’ Λ = 0.972, *F* (4, 181) = 1.31, *p* = 0.267, *ηp*^2^ = 0.028], and none of the univariate tests was significant (all *p* ≥ 0.277; *ηp*^2^ ≤ 0.006). The sex × ethnicity interaction was not significant in either model (*p* = 0.137 and 0.642).

As shown in Table 6, girls tended to report slightly more LPA (*M*_girls_ = 6.29 ± 5.77 vs. *M*_boys_ = 6.04 ± 5.82; *p* = 0.293, *d* = 0.04), screen-based SB (*M*_girls_ = 2.45 ± 1.90 vs. *M*_boys_ = 2.01 ± 1.67; *p* = 0.198, *d* = 0.24), and non-screen-based SB (*M*_girls_ = 6.51 ± 4.69 vs. *M*_boys_ = 4.35 ± 2.93; *p* = 0.240, *d* = 0.52), whereas boys reported slightly more MVPA than girls (*M*_boys_ = 32.01 ± 24.08 vs. *M*_girls_ = 29.86 ± 23.89; *p* = 0.618, *d* = 0.09). For EF, boys showed a tendency toward higher EF scores, indicating more parent-reported difficulties, in working memory (*M*_boys_ = 16.30 ± 5.24 vs. *M*_girls_ = 13.01 ± 6.67; *p* = 0.277, *d* = 0.53), planning (*M*_boys_ = 6.98 ± 2.95 vs. *M*_girls_ = 5.54 ± 3.30; *p* = 0.446, *d* = 0.45), regulation (*M*_boys_ = 9.42 ± 3.15 vs. *M*_girls_ = 8.83 ± 3.89; *p* = 0.580, *d* = 0.16), and inhibition (*M*_boys_ = 10.80 ± 3.17 vs. *M*_girls_ = 9.65 ± 4.03; *p* = 0.471, *d* = 0.31). These descriptive patterns were not significant after controlling for age and BMI and should be interpreted with caution.

**Figure 2:**
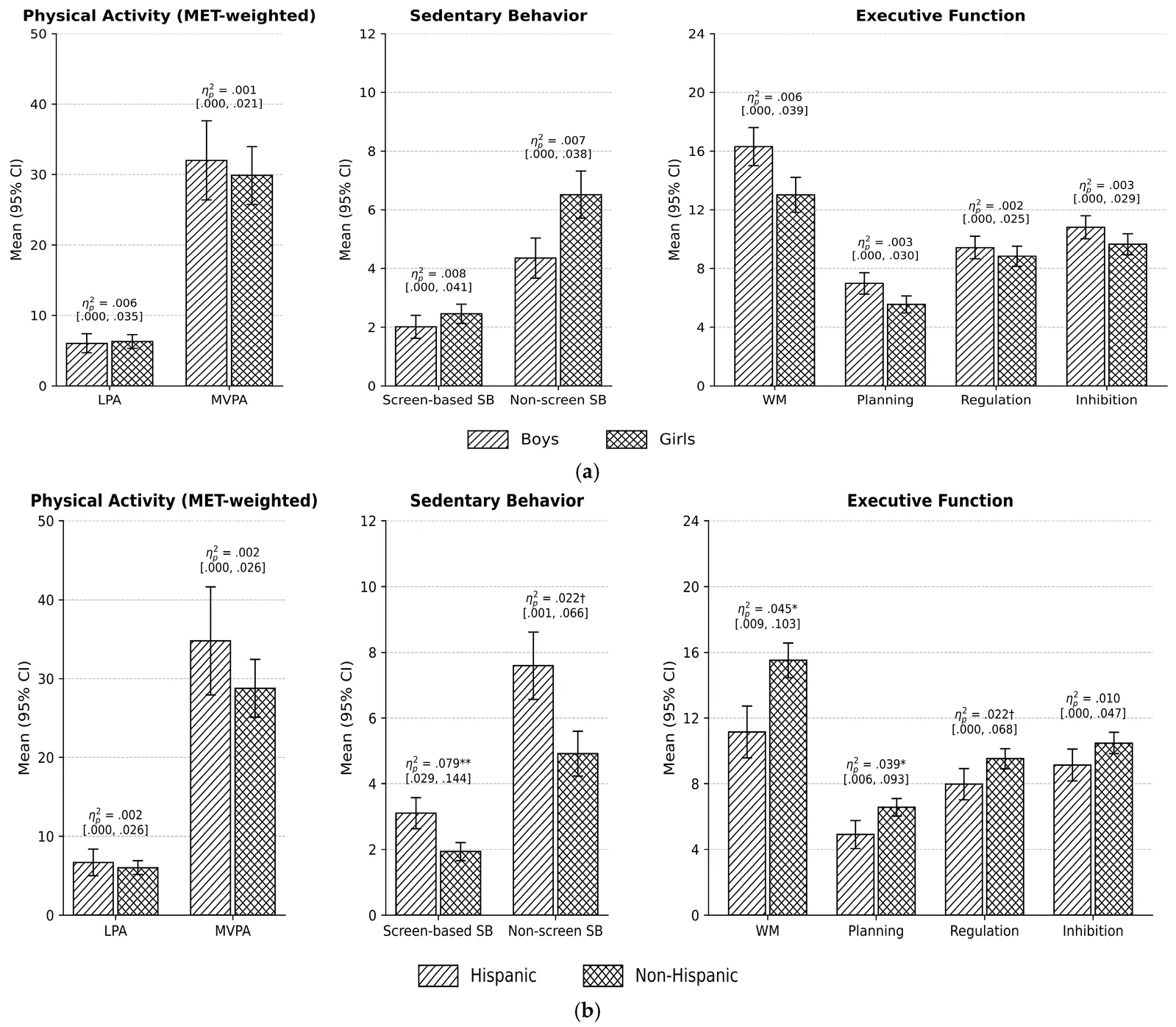
(**a**) Sex Differences in Physical Activity and Executive Function. (**b**) Ethnic Differences in Physical Activity and Executive Function. Note: Means (95% CI) of outcomes by sex and ethnicity. Values above bars are partial eta squared (η*ρ*^2^) with 95% confidence intervals. LPA = light physical activity; MVPA = moderate-to-vigorous physical activity; SB = sedentary behavior; WM = working memory. † *p* < 0.10; * *p* < 0.05; ** *p* < 0.01.

The first MANCOVA model indicated a significant multivariate effect of ethnicity [Wilks’ Lambda = 0.904, *F* (4, 197) = 5.24, *p* < 0.001, *ηp*^2^ = 0.096] after controlling for youth’s age and BMI (Figure 2b). Univariate tests showed a significant ethnic difference in screen-based SB *F* (1, 200) = 17.27, *p* < 0.001, *p* = 0.001, *ηp*^2^ = 0.079) and non-screen-based SB *F* (1, 200) = 4.53, *p* = 0.034, *ηp*^2^ = 0.022].

The second MANCOVA model revealed a significant main effect of ethnicity [Wilks’ Lambda = 0.944, *F* (4, 181) = 2.70, *p* = 0.032, *ηp*^2^ = 0.056] on EF (i.e., working memory, planning, regulation, inhibition) after controlling for age and BMI (Figure 2b). Univariate tests showed a significant ethnic difference in working memory *F* (1, 184) = 8.70, *p* = 0.004, *p*_Holm_ = 0.014, *ηp*^2^ = 0.045), planning *F* (1, 184) = 7.38, *p* = 0.007, *p_Holm_* = 0.022, *ηp*^2^ = 0.039), and regulation *F* (1, 184) = 4.10, *p* = 0.044, *p_Holm_* = 0.089, *ηp*^2^ = 0.022). As shown in Table 6, Hispanic youth reported significantly more screen-based SB than non-Hispanic youth (M_Hispanic_ = 3.10 ± 1.90 vs. M_non-Hispanic_ = 1.93 ± 1.68; *p* < 0.001, d = 0.67). Hispanic youth also tended to report more non-screen-based SB (M_Hispanic_ = 7.59 ± 4.08 vs. M_non-Hispanic_ = 4.91 ± 4.10; *p* = 0.034, *d* = 0.65). Hispanic youth had significantly lower EF scores, indicating fewer EF difficulties, in working memory (M_Hispanic_ = 11.14 ± 6.06 vs. M_non-Hispanic_ = 15.51 ± 6.09; *p* = 0.004, *d* = 0.72), planning (M_Hispanic_ = 4.90 ± 3.29 vs. M_non-Hispanic_ = 6.56 ± 3.11; *p* = 0.007, *d* = 0.52), and regulation (M_Hispanic_ = 7.97 ± 3.65 vs. M_non-Hispanic_ = 9.52 ± 3.57; *p* = 0.044, *d* = 0.43).

## 4. Discussion

The purpose of this study was to examine how multiple ecological environmental factors, including neighborhood safety and crime, community walkability, home media environment, socioeconomic environment, and geographic environment, were associated with physical activity (PA), sedentary behavior (SB), and executive function (EF) among youth; and to test whether PA served as a mediating mechanism linking ecological environment to EF. Guided by the Social Ecological Model [11,12], the findings suggest that cognitive functioning among youth is not determined by individual-level characteristics alone, but is systematically embedded within broader home, neighborhood, socioeconomic, and geographic contexts. Across a comprehensive data analysis, ecological environmental factors demonstrated meaningful associations with PA, SB, and EF, with particularly consistent evidence for neighborhood safety/crime as a critical correlate of EF outcomes.

The pattern of associations among ecological environmental factors and PA and SB support that health behavior may be shaped simultaneously by contextual forces operating across multiple ecological levels [13]. The findings indicate that daily movement profiles among youth vary systematically across home and neighborhood contexts, and that the home media environment and perceived conditions in the surrounding community were both meaningful correlates of PA. Prior research has documented that the density of screen-based equipment in the home is positively associated with adolescent screen time and inversely associated with accelerometer-measured PA [60,61]. In contrast, youth with more types of media devices in their bedroom reported more MVPA in this study, and the home media environment was not associated with SB. Bedroom media availability may partly reflect household resources that also support participation in organized sport and recreation, and the measure captured device availability rather than use; this unexpected association should be replicated with objective measures of media use and PA.

Youth who perceive their neighborhoods as unsafe are less likely to walk, cycle, or engage in unstructured outdoor recreation, a behavioral suppression that has downstream consequences for cumulative daily PA. Geographic environment, captured here through residential location indicators, further contextualized these socioeconomic effects, consistent with the well-established concentration of resource constraints and activity-limiting conditions in specific residential areas [20]. These findings underscore that SB among youth is not simply a product of individual choice or motivation but is structured by the material and infrastructural conditions of their residential environments.

Neighborhood safety and crime and community walkability demonstrated the most consistent associations with EF in this study. These findings extend a growing literature documenting the direct effects of neighborhood conditions and built environment with adolescent cognitive development. Using data from the Adolescent Brain Cognitive Development (ABCD) Study, Lindsley et al. [22] found that perceived neighborhood safety over and above objective crime rates were associated with better working memory and reading comprehension in adolescents. Walkable community design promotes active transportation, outdoor play, and spontaneous PA, all of which contribute to prefrontal activation and neuroplastic adaptation [7]. Caetano et al. [18], using GIS-derived walkability indices and kernel density estimation, found that adolescents in higher-walkability neighborhoods were significantly more likely to meet PA guidelines, reinforcing the behavioral pathway that may partially link walkability to EF. From a developmental perspective, the consistent ecological associations with EF may carry clinical implications. Environmental stressors such as unsafe neighborhoods, limited walkable infrastructure, and socioeconomic resource constraints, may converge to restrict independent mobility, increase allostatic load, reduce opportunities for cognitively enriching activity, and thereby impair the regulatory processes that EF subsumes. Future research should test whether considering neighborhood safety and walkability as contextual factors, alongside individual behavioral counseling, improves the assessment of cognitive and behavioral concerns in pediatric and adolescent care.

The social-ecological pathway from the ecological environmental factors to EF by testing the mediation role of PA in this relationship is the unique contribution of this study. The structural model showed a strong direct association between the latent ecological environmental factor and EF, although the indirect effect of ecological environment on EF through PA was small in magnitude, and the mediation hypothesis was not supported in this sample. The non-significant indirect effect does not support PA as the pathway linking the environment to EF in this sample. Other pathways, such as stress, sleep, and cognitive stimulation, have been proposed in prior work [22,23,62], but they were not tested in this study. PA research conducted in naturalistic environmental contexts has demonstrated that the cognitive benefits of PA are moderated by environmental exposures including green space access, air quality, and sensory conditions, such that the same PA dose may produce different EF outcomes depending on the environmental context in which it occurs [63]. These considerations collectively suggest that PA, while an important health-promoting behavior, may not be the dominant mediating mechanism linking ecological environment to EF in cross-sectional community samples. Future studies should test more refined models incorporating objective PA measures, 24-h movement compositions, fitness indices, sleep quality, and stress-related biomarkers to decompose the relative contribution of behavioral versus psychosocial versus neurobiological pathways. Because walkability and neighborhood safety/crime loaded strongly on the environmental factor whereas socioeconomic, geographic, and home media environments loaded weakly, the latent factor primarily reflects parents’ perceptions of neighborhood safety and walkability. The sensitivity analysis with separate environmental domains supported that the association with EF was carried mainly by neighborhood safety/crime and walkability. Conclusions about a broad “ecological environment” should therefore be interpreted with caution, and future studies with larger and more geographically diverse samples should test whether these domains are better modeled separately or as a formative composite.

The MANCOVA analyses revealed significant ethnic differences in screen-based SB, non-screen-based SB and multiple EF components after controlling for age and BMI. Hispanic youth demonstrated higher overall SB and lower working memory, planning, and regulation scores relative to non-Hispanic peers, while no significant sex differences were observed in this study. These findings are consistent with prior evidence of ethnic disparities in both screen time exposure and EF performance during childhood and early adolescence. Zhang and colleagues [64] documented longitudinal ethnic disparities in EF and physical activity between Hispanic and non-Hispanic children from kindergarten through third grade, with Hispanic children demonstrating lower psychosocial and physical functioning trajectories over time. Nagata et al. [65], analyzing data from the ABCD Study, found that Black and Latinx/Hispanic adolescents were more likely to experience problematic screen use, attributing these disparities in part to neighborhood safety perceptions, reduced access to outdoor recreational spaces, and differential parental supervision time related to occupational demands.

The ethnic patterns observed in the present study should not be interpreted as reflecting group-level individual differences in ability or motivation. Differential ecological exposure is one possible explanation. Hispanic families in this sample reported lower household income than non-Hispanic families, but they did not report greater neighborhood safety concerns, and this study did not test whether environmental factors accounted for the ethnic differences. Perceptions of neighborhood safety have been specifically associated with increased screen time and decreased PA among minority youth [65] suggesting that screen time disparities may reflect behavioral adaptation to constrained neighborhood conditions rather than individual health behavior failure; this hypothesis requires direct testing. Socioeconomic status and race/ethnicity in the United States function as proxies for differential exposure to ecological risk and resource conditions, and healthcare and public health interventions must address these structural determinants rather than targeting individual behavior in isolation.

If confirmed in longitudinal research, these findings would have implications for pediatric and adolescent healthcare practice. Healthcare providers conducting well-child visits and adolescent health assessments could inquire about residential neighborhood conditions, including perceived safety, access to walkable streets and parks, availability of recreational programs, and household screen media environments, in addition to individual PA and dietary behaviors. Ecological screening of this kind aligns with the American Academy of Pediatrics’ advocacy for social determinants of health screening in clinical settings and reflects the growing recognition that neighborhood and community-level factors are as clinically relevant as individual behavioral factors in shaping adolescent cognitive and physical health outcomes [12].

At the community and policy level, the findings reinforce the argument that youth health promotion is an intersectoral responsibility requiring coordination among clinical healthcare, public health, education, transportation planning, and civil engineering. Community walkability is determined by street connectivity, pedestrian infrastructure design, land use mix, and traffic management; and it is not a healthcare variable in the traditional sense. Investment in pedestrian infrastructure, traffic-calming design, mixed-use zoning, and safe active transportation routes may represent a population-level lever for improving youth physical activity and brain health. Restricting household screen time through family-level education alone is unlikely to be sufficient when outdoor play and active transportation are limited by neighborhood safety concerns and infrastructural barriers. Future interventions could combine family media literacy and rule-setting with community-level provision of safe, accessible, and appealing alternatives for after-school activity, including supervised recreation programs, community lighting improvements, and school-community recreation partnerships.

These findings should be interpreted as associations rather than causal effects. Because the data were cross-sectional, the results cannot establish whether environmental factors preceded differences in PA or EF. The practical implications described should likewise be viewed as hypotheses for future longitudinal and intervention research rather than as evidence-based recommendations. Several limitations constrain the interpretation and generalizability of the findings. All environmental exposures and child outcomes were reported by a single parent, and the environmental items were adapted from existing instruments without validation in this population. The home media environment measure captured device availability in the bedroom but not media use, and the neighborhood safety scale combined perceived barriers with perceived crime rather than assessing victimization. The parent version of the NEWS-Y has been evaluated for test–retest reliability among parents of children aged 5–11 years and parents of adolescents aged 12–18 years [39,40]; however, the selected and adapted items used here have not been validated in this population.

Although the SEM specified a theoretically guided directional pathway from ecological environment through PA to EF, temporal ordering and directionality cannot be confirmed from these data. The null result is therefore inconclusive rather than evidence that PA does not link the environment to EF. Similarly, the ethnic differences in SB and EF may reflect differential ecological exposure, but this study did not test whether environmental factors accounted for these differences, so this explanation remains a hypothesis. Further mediation analyses examining whether environmental factors account for ethnic differences were not pursued because the environmental factor was not unidimensional, measurement invariance across ethnic groups could not be established with the available subgroup sizes, and power to detect indirect effects was limited.

In addition, the home media environment scale showed modest internal consistency. Because the scale is an inventory of distinct bedroom devices rather than a measure of a single underlying construct, modest consistency was expected; however, the resulting measurement error may have attenuated its associations with outcomes. The sample also provided limited power to detect small indirect effects, so the null mediation finding should be interpreted as inconclusive rather than as evidence of no mediation. Future studies should model ecological levels as separate predictors or as formative composites and test these specifications in larger, longitudinal samples. BMI was entered as a continuous covariate rather than as age- and sex-specific BMI percentiles or z-scores, and future studies should use BMI-for-age standards. Ecological environmental factors were similarly assessed through questionnaire-based measures rather than objective GIS-derived indicators of walkability, crime, or residential location, limiting the specificity of environmental characterization. Future studies should incorporate objective, geospatially derived environmental measures alongside device-based 24-h movement behavior monitoring and longitudinal designs to test the mediation hypothesis more rigorously.

## 5. Conclusions

This study provides evidence that ecological environmental factors are meaningfully associated with youth PA and cognitive health, with neighborhood safety and crime and community walkability demonstrating the most consistent associations with EF outcomes. Socioeconomic environment was the primary predictor of SB, while home media environment and neighborhood safety/crime played an important role for active lifestyle. In the SEM, the environmental factor was directly associated with EF, but the indirect pathway through PA was not supported; whether other behavioral or psychosocial pathways link the environment to EF was not tested and remains a question for longitudinal research. Ethnic differences were observed in screen-based SB and EF, with Hispanic youth reporting more screen-based SB but fewer parent-reported EF difficulties; whether these differences reflect differential ecological exposure is a hypothesis that this study did not test. Taken together, these cross-sectional findings support the application of the Social Ecological Model to youth cognitive health and suggest that future multilevel, equity-focused interventions could address individual health behaviors and the community, neighborhood, and infrastructural conditions that shape them.

## Figures and Tables

**Table 1:** Descriptive Statistics of Study Variables.

Variables	Range	M (SD)
Age	7–18	15.52 (3.19)
BMI	11.98–48.82	23.80 (6.06)
Socioeconomic Environment (Household Income)		
	<30,000	36 (13.3)
	31,000–49,000	111 (41.0)
	50,000–69,000	54 (19.9)
	>70.000	70 (25.8)
Geographic Environment	1–4	1.03 (0.26)
Home Media Environment	7–14	10.05 (1.77)
Community Walkability	5–20	11.50 (2.52)
Neighborhood Safety/crime	0–36	19.19 (5.37)
Physical Activity Behaviors		
LPA	0–21	6.20(5.76)
MVPA	0–98	29.92 (23.74)
Screen-based SB	0–6	2.28 (1.77)
Non-Screen-Based SB	0–24	5.54 (4.14)
Executive function	0–84	39.30 (15.167)
Working Memory	0–34	14.28 (6.21)
Planning	0–16	6.13 (3.10)
Regulation	0–20	8.82 (3.59)
Inhibition	0–20	10.03 (3.78)

Note: LPA = mild frequency × 3 METs; MVPA = (moderate frequency × 5 METs) + (vigorous frequency × 9 METs). LPA = light physical activity; MVPA = moderate-to-vigorous physical activity; SB = sedentary behavior; socioeconomic environment is represented by household income.

**Table 2:** Bivariate Correlations Among Study Variables.

Variable	1	2	3	4	5	6	7	8	9	10	11	12	13
1. Geographic environment	—												
2. Socioeconomic environment	−0.101	—											
3. Home media environment	0.005	0.086	—										
4. Neighborhood safety/crime	0.094	−0.152 *	−0.159 *	—									
5. Community walkability	0.021	−0.302 **	−0.103	0.388 **	—								
6. Light PA	0.019	−0.009	−0.118	−0.120	−0.071	—							
7. MVPA	−0.049	0.021	−0.244 **	−0.230 **	−0.128	0.636 **	—						
8. Screen-based SB	0.057	0.059	0.032	−0.163 *	−0.233 **	−0.138 *	−0.154 *	—					
9. Non-screen-based SB	0.033	0.022	−0.050	−0.049	−0.048	0.001	−0.005	0.611 **	—				
10. Working memory	0.097	−0.134 *	−0.089	0.399 **	0.312 **	−0.092	−0.161 *	−0.087	−0.113	—			
11. Planning	0.182 **	−0.182 **	−0.140 *	0.422 **	0.381 **	−0.117	−0.174 *	−0.101	−0.084	0.854 **	—		
12. Regulation	0.148 *	0.003	−0.056	0.363 **	0.156 *	−0.122	−0.278 **	0.157 *	0.080	0.721 **	0.660 **	—	
13. Inhibition	0.126	−0.208 **	−0.123	0.367 **	0.311 **	−0.080	−0.139 *	−0.005	0.014	0.745 **	0.743 **	0.680 **	—

Note: Values are Pearson correlation coefficients. Column numbers correspond to the numbered variables in the first column. PA = physical activity; MVPA = moderate-to-vigorous physical activity; SB = sedentary behavior. * *p* < 0.05. ** *p* < 0.01.

**Table 3:** Hierarchical Regression Model Results for Physical Activity and Executive Function.

Predictors	Physical Activity						Sedentary Behavior						Executive Function					
	R^2^	B	SE	95% CI	β	*p*	R^2^	B	SE	95% CI	β	*p*	R^2^	B	SE	95% CI	β	*p*
Step 1	0.009						0.116 **						0.128 **					
Sex		−5.028	4.672	[−14.251, 4.194]	−0.088	0.283		1.504	0.828	[−0.131, 3.138]	0.136	0.071		−3.796	2.404	[−8.543, 0.952]	−0.122	0.116
Age		0.899	1.093	[−1.259, 3.056]	0.067	0.412		0.684	0.192	[0.304, 1.064]	0.267 **	<0.001		−2.078	0.544	[−3.152, −1.004]	−0.294 **	<0.001
Ethnicity		0.714	1.426	[−2.100, 3.527]	0.038	0.617		0.052	0.257	[−0.455, 0.559]	0.014	0.841		−0.437	0.751	[−1.920, 1.046]	−0.044	0.561
Step 2	0.204 **						0.181 **						0.274 **					
Sex		−4.783	4.329	[−13.329, 3.763]	−0.083	0.271		1.559	0.821	[−0.061, 3.180]	0.141	0.059		−2.946	2.266	[−7.423, 1.530]	−0.094	0.196
Age		0.206	1.031	[−1.830, 2.243]	0.015	0.842		0.748	0.194	[0.365, 1.132]	0.292 **	<0.001		−1.846	0.520	[−2.874, −0.819]	−0.261 **	<0.001
Ethnicity		0.392	1.367	[−2.307, 3.092]	0.021	0.775		0.025	0.266	[−0.500, 0.549]	0.007	0.926		0.464	0.731	[−0.980, 1.907]	0.046	0.527
SES		−0.814	2.174	[−5.105, 3.478]	−0.028	0.709		0.863	0.415	[0.043, 1.683]	0.156 *	0.039		−0.714	1.137	[−2.961, 1.533]	−0.047	0.531
Geographic		−10.905	8.359	[−27.408, 5.598]	−0.093	0.194		2.605	1.639	[−0.630, 5.839]	0.113	0.114		8.984	4.362	[0.367, 17.600]	0.145 *	0.041
Home media		−4.552	1.140	[−6.802, −2.302]	0.288 **	<0.001		−0.213	0.217	[−0.641, 0.216]	−0.071	0.328		0.222	0.616	[−0.995, 1.439]	0.026	0.719
Walkability		−0.924	0.871	[−2.645, 0.796]	−0.084	0.290		−0.264	0.171	[−0.602, 0.073]	−0.122	0.124		0.951	0.475	[0.012, 1.890]	0.155 *	0.047
Safety/crime		−1.764	0.402	[−2.558, −0.969]	−0.340 **	<0.001		−0.019	0.077	[−0.172, 0.133]	−0.019	0.801		0.753	0.217	[0.323, 1.182]	0.265 **	<0.001
ΔR^2^	0.195 **						0.065 *						0.146 **					
ΔF	8.19 **						2.76 *						6.26 **					

Note. SES = socioeconomic environment; Home media = home media environment; Walkability = community walkability; Safety/crime = neighborhood safety/crime. Physical activity = Godin–Shephard leisure-time activity index (LPA + MVPA); sedentary behavior = total daily sitting time; higher executive function scores indicate greater difficulties. * *p* < 0.05. ** *p* < 0.01.

**Table 4:** (**a**) Hierarchical Regression Model Results for Working Memory and Planning. (**b**) Hierarchical Regression Model Results for Regulation and Inhibition.

(a)															
Predictors	Working Memory								Planning						
	R^2^		B	SE	95% CI		β	*p*	R^2^		B	SE	95% CI	β	*p*
Step 1	0.160 **								0.132 **						
Sex			−1.826	0.945	[−3.692, 0.040]		−0.142	0.055			−0.911	0.465	[−1.828, 0.007]	−0.144	0.052
Age			−0.907	0.215	[−1.332, −0.482]		−0.311 **	<0.001			−0.336	0.105	[−0.542, −0.129]	−0.237 **	0.002
Ethnicity			−0.338	0.296	[−0.923, 0.246]		−0.081	0.255			−0.293	0.145	[−0.579, −0.006]	−0.143 *	0.045
Step 2	0.289 **								0.305 **						
Sex			−1.753	0.897	[−3.524, 0.019]		−0.136	0.053			−0.754	0.429	[−1.601, 0.092]	−0.119	0.080
Age			−0.821	0.208	[−1.231, −0.410]		−0.281 **	<0.001			−0.304	0.099	[−0.499, −0.110]	−0.215 **	0.002
Ethnicity			−0.060	0.292	[−0.636, 0.516]		−0.014	0.838			−0.075	0.140	[−0.351, 0.200]	−0.037	0.590
SES			−0.417	0.459	[−1.323, 0.489]		−0.065	0.365			−0.340	0.218	[−0.770, 0.089]	−0.109	0.120
Geographic			2.341	1.787	[−1.187, 5.869]		0.088	0.192			1.685	0.862	[−0.016, 3.387]	0.128	0.052
Home media			0.225	0.243	[−0.254, 0.703]		0.064	0.356			−0.023	0.115	[−0.250, 0.204]	−0.013	0.843
Walkability			0.355	0.191	[−0.022, 0.731]		0.138	0.064			0.224	0.090	[0.046, 0.403]	0.182 *	0.014
Safety/crime			0.302	0.085	[0.133, 0.470]		0.257 **	<0.001			0.141	0.042	[0.058, 0.224]	0.242 **	<0.001
ΔR^2^	0.129 **								0.173 **						
ΔF	6.06 **								8.54 **						
(b)															
Predictors	Regulation								Inhibition						
	R^2^	B	SE		95% CI	β	*p*		R^2^	B	SE		95% CI	β	*p*
Step 1	0.074 **								0.070 **						
Sex		−0.119	0.568		[−1.239, 1.002]	−0.016	0.834			−0.579	0.595		[−1.752, 0.595]	−0.075	0.332
Age		−0.445	0.126		[−0.693, −0.196]	−0.269 **	<0.001			−0.409	0.133		[−0.672, −0.146]	−0.236 **	0.002
Ethnicity		0.062	0.179		[−0.292, 0.416]	0.025	0.729			0.084	0.186		[−0.283, 0.452]	0.033	0.652
Step 2	0.204 **								0.226 **						
Sex		−0.041	0.543		[−1.113, 1.031]	−0.005	0.940			−0.349	0.559		[−1.453, 0.754]	−0.045	0.533
Age		−0.327	0.123		[−0.569, −0.085]	−0.198 **	0.008			−0.366	0.128		[−0.618, −0.113]	−0.211 **	0.005
Ethnicity		0.234	0.178		[−0.119, 0.586]	0.095	0.192			0.324	0.182		[−0.035, 0.683]	0.129	0.077
SES		0.379	0.274		[−0.163, 0.921]	0.102	0.169			−0.562	0.283		[−1.120, −0.004]	−0.146 *	0.049
Geographic		3.094	1.085		[0.953, 5.235]	0.200 **	0.005			1.641	1.115		[−0.560, 3.842]	0.103	0.143
Home media		0.032	0.144		[−0.253, 0.317]	0.016	0.826			−0.037	0.150		[−0.333, 0.260]	−0.017	0.808
Walkability		0.020	0.113		[−0.203, 0.244]	0.014	0.858			0.223	0.117		[−0.009, 0.455]	0.147	0.059
Safety/crime		0.211	0.051		[0.110, 0.313]	0.313 **	<0.001			0.163	0.054		[0.057, 0.269]	0.232 **	0.003
ΔR^2^	0.130 **								0.156 **						
ΔF	5.65 **								6.83 **						

Note: * *p* < 0.05; ** *p* < 0.01.

**Table 5:** Model Fit Indices and Structural Path Coefficients of the SEM.

	χ^2^/B	df/SE	NFI/CR	TLI/β	CFI/*p*	RMSEA	90% CI [LL, UL]
Model Fit Indices							
Structural Model (SEM)	42.321	25	0.904	0.922	0.956	0.050	[0.021, 0.075]
Structural Path Coefficients							
Path (Predictor → Outcome)	B	SE	CR	β	p		
Direct Paths							
Environmental Factor → Physical Activity	5.377	2.971	1.810	0.207	0.070		
Physical Activity → Executive Function	−0.097	0.137	−0.708	−0.052	0.479		
Environmental Factor → Executive Function	−27.960	8.732	−3.202	−0.575 **	0.001		
Indirect Path							
Environmental Factor → Physical Activity → Executive Function	−0.521	—	—	−0.011 ^a^	[−2.60, 1.03] ^b^		
Measurement Model: Environmental Factor							
Socioeconomic environment	1.000 ^c^	—	—	0.307	—		
Geographic environment	−0.110	0.067	−1.640	−0.132	0.101		
Home media environment	1.022	0.543	1.883	0.180	0.060		
Community walkability	−4.838	1.346	−3.595	−0.598 **	<0.001		
Neighborhood safety/crime	−11.763	3.645	−3.227	−0.682 **	0.001		
Measurement Model: Physical Activity Factor							
Moderate PA (MET-weighted)	1.000 ^c^	—	—	0.908	—		
Vigorous PA (MET-weighted)	1.491	0.137	10.906	0.722 **	<0.001		
Light PA (MET-weighted)	0.529	0.047	11.281	0.746 **	<0.001		

Note. Estimates were obtained with full-information maximum likelihood. B = unstandardized coefficient; SE = standard error; CR = critical ratio; β = standardized coefficient. ^a^ Standardized indirect effect. ^b^ Bias-corrected bootstrap 95% confidence interval for the unstandardized indirect effect, based on 5000 bootstrap samples; the interval includes zero, indicating a nonsignificant indirect effect. ^c^ Loading fixed to 1.000 to identify the latent factor; *SE*, CR, and *p* were therefore not estimated. ** *p* < 0.01.

**Table 6:** Multivariate Analysis on Sex and Ethnicity.

Variables	Sex						Ethnicity					
	Boys *M* (*SD*)	Girls *M* (*SD*)	*F*	*p*	*p*_Holm_	η_p_^2^	Hispanic *M* (*SD*)	Non-Hispanic *M* (*SD*)	*F*	*p*	*p*_Holm_	η_p_^2^
Model 1: Physical activity and sedentary behavior												
LPA(MET-weighted)	6.04 (5.82)	6.29 (5.77)	1.114	0.293	0.794	0.006	6.66 (6.77)	6.00 (5.28)	0.471	0.493	0.964	0.002
MVPA(MET-weighted)	32.01 (24.08)	29.86 (23.89)	0.249	0.618	0.794	0.001	34.77 (27.39)	28.76 (22.03)	0.496	0.482	0.964	0.002
Screen-based SB	2.01 (1.67)	2.45 (1.90)	1.665	0.198	0.794	0.008	3.10 (1.90)	1.93 (1.68)	17.265	<0.001 **	<0.001 **	0.079
Non-screen-based SB	4.35 (2.93)	6.51 (4.69)	1.391	0.240	0.794	0.007	7.59 (4.08)	4.91 (4.10)	4.533	0.034 *	0.103	0.022
Model 2: Executive function												
WM	16.30 (5.24)	13.01 (6.67)	1.190	0.277	1.000	0.006	11.14 (6.06)	15.51 (6.09)	8.696	0.004 **	0.014 *	0.045
Planning	6.98 (2.95)	5.54 (3.30)	0.584	0.446	1.000	0.003	4.90 (3.29)	6.56 (3.11)	7.376	0.007 **	0.022 *	0.039
Regulation	9.42 (3.15)	8.83 (3.89)	0.307	0.580	1.000	0.002	7.97 (3.65)	9.52 (3.57)	4.101	0.044 *	0.089	0.022
Inhibition	10.80 (3.17)	9.65 (4.03)	0.521	0.471	1.000	0.003	9.14 (3.75)	10.47 (3.74)	1.885	0.171	0.171	0.010

Note. Values are observed means and standard deviations. Each model was a 2 (sex) × 2 (ethnicity) MANCOVA adjusted for age and BMI; univariate *F* tests are follow-up ANCOVAs. Ethnicity = Hispanic vs. non-Hispanic. LPA and MVPA are MET-weighted. Higher EF scores indicate greater difficulties. PA = physical activity; LPA = light PA; MVPA = moderate-to-vigorous PA; SB = sedentary behavior; WM = working memory. * *p* < 0.05. ** *p* < 0.01.

## Data Availability

The data that support the findings of this study are available from the corresponding author upon reasonable request. The data is not publicly available due to privacy and ethical considerations related to the study participants.

## Supplementary Materials

The following supporting information can be downloaded at: https://www.mdpi.com/article/10.3390/healthcare14213737/s1, Table S1. Sensitivity Analysis: Structural Equation Model With Environmental Domains as Separate Predictors.
