# Supplementary material for: Built and Social Environmental Correlates of Brain and Behavioral Health Among Youth
Source: Healthcare (Basel). 2026 Nov 3;14(21):3737. doi: 10.3390/healthcare14213737 (PMC13762959; doi:10.3390/healthcare14213737)
Supplement: Supplementary file 1 [file healthcare-14-03737-s001.zip › healthcare-4619015-supplementary.pdf]

Table S1. *Sensitivity Analysis: Structural Equation Model With Environmental Domains as Separate Predictors*

	→ Physical activity (latent)				→ Executive function				Indirect effect via PA	
Predictor	*B*	*SE*	β	*p*	*B*	*SE*	β	*p*	*B*	95% CI
Socioeconomic environment	−0.100	0.580	−0.01	0.863	−0.286	0.971	−0.02	0.768	0.018	[−0.245, 0.305]
Geographic environment (RUCA)	−0.305	1.911	−0.01	0.873	5.716	3.114	0.10	0.066	0.053	[−0.818, 1.000]
Home media environment	**−1.326**	**0.321**	**−0.29****	**<0.001**	−0.292	0.585	−0.03	0.618	0.232	[−0.107, 0.632]
Community walkability	−0.202	0.246	−0.06	0.411	**1.191**	**0.424**	**0.20****	**0.005**	0.035	[−0.062, 0.181]
Neighborhood safety/crime	**−0.351**	**0.114**	**−0.23****	**0.002**	**0.840**	**0.199**	**0.30****	**<0.001**	0.061	[−0.028, 0.178]
Physical activity	—	—	—	—	−0.175	0.132	−0.09	0.184	—	—

Note. Full-information maximum likelihood estimates. The five environmental domains were entered as correlated exogenous predictors; physical activity was a latent factor indicated by MET-weighted moderate, vigorous, and light PA (moderate loading fixed to 1). Model fit: $\chi^2(12) = 10.61$, $p = 0.563$, CFI = 1.00, TLI = 1.00, RMSEA = 0.000, 90% CI [0.000, 0.055]. $R^2 = 0.13$ for PA and 0.22 for EF. Indirect effects were tested with Monte Carlo 95% CIs. Higher home media scores indicate fewer types of media devices in the bedroom; higher EF scores indicate greater difficulties. *B* = unstandardized coefficient; *SE* = standard error; β = standardized coefficient. *$p < 0.05$. **$p < 0.01$
